# Supplementary material for: MetaRibo-Seq measures translation in microbiomes
Source: Nat Commun. 2020 Jun 29;11:3268. doi: 10.1038/s41467-020-17081-z (PMC7324362; doi:10.1038/s41467-020-17081-z)
Supplement: Supplementary file 10 — Supplementary Data 7 [file 41467_2020_17081_MOESM10_ESM.zip › File2/Confidence_VeryHigh_Taxonomy/38837_out.krona.html]

Javascript must be enabled to view this page.

members
magnitude
magnitudeUnassigned
count
unassigned
taxon
rank

38837\_out

16

16
superkingdom
2

1239
16
phylum

16
class
186801

186802
order
16

family
12
31979

1

SRS148970\_contig\_number\_contig-100\_18371.18372
1485
12
genus

2293041

SRS015890\_contig\_number\_14350SRS018984\_contig\_number\_14563SRS050422\_contig\_number\_27367
3
species

2293024
species

SRS016267\_contig\_number\_contig-100\_52.83418SRS019397\_contig\_number\_11950SRS019787\_contig\_number\_9850SRS054590\_contig\_number\_1641SRS074670\_contig\_number\_contig-100\_9152.9152SRS142503\_contig\_number\_contig-100\_5785.225530SRS142712\_contig\_number\_88SRS143148\_contig\_number\_9498
8

family
2
186806

1730
genus
2

species

SRS019445\_contig\_number\_contig-100\_3557.155077SRS054905\_contig\_number\_8193
2
39485

family
2
186803


SRS075078\_contig\_number\_23627SRS105153\_contig\_number\_18973
2
species
39491
